# Neural correlates of body and face perception following bilateral destruction of the primary visual cortices

**DOI:** 10.3389/fnbeh.2014.00030

**Published:** 2014-02-13

**Authors:** Jan Van den Stock, Marco Tamietto, Minye Zhan, Armin Heinecke, Alexis Hervais-Adelman, Lore B. Legrand, Alan J. Pegna, Beatrice de Gelder

**Affiliations:** ^1^Brain and Emotion Laboratory Leuven, Division of Psychiatry, Department of Neurosciences, KU LeuvenLeuven, Belgium; ^2^Cognitive and Affective Neuroscience Laboratory, and CoRPS - Center of Research on Psychology in Somatic Diseases - Tilburg UniversityTilburg, Netherlands; ^3^Department of Psychology, University of TorinoTorino, Italy; ^4^Department of Cognitive Neuroscience, Faculty of Psychology and Neuroscience, Maastricht UniversityMaastricht, Netherlands; ^5^Brain InnovationMaastricht, Netherlands; ^6^Faculty of Psychology and Educational Science, University of GenevaGeneva, Switzerland; ^7^Laboratory of Experimental Neuropsychology, Neuropsychology Unit and Department of Neurology, Geneva University HospitalsGeneva, Switzerland

**Keywords:** residual vision, V1, EBA, ventral stream, cerebellum, orbitofrontal cortex, amygdala, insula

## Abstract

Non-conscious visual processing of different object categories was investigated in a rare patient with bilateral destruction of the visual cortex (V1) and clinical blindness over the entire visual field. Images of biological and non-biological object categories were presented consisting of human bodies, faces, butterflies, cars, and scrambles. Behaviorally, only the body shape induced higher perceptual sensitivity, as revealed by signal detection analysis. Passive exposure to bodies and faces activated amygdala and superior temporal sulcus. In addition, bodies also activated the extrastriate body area, insula, orbitofrontal cortex (OFC) and cerebellum. The results show that following bilateral damage to the primary visual cortex and ensuing complete cortical blindness, the human visual system is able to process categorical properties of human body shapes. This residual vision may be based on V1-independent input to body-selective areas along the ventral stream, in concert with areas involved in the representation of bodily states, like insula, OFC, and cerebellum.

## Introduction

**Figure 1:**
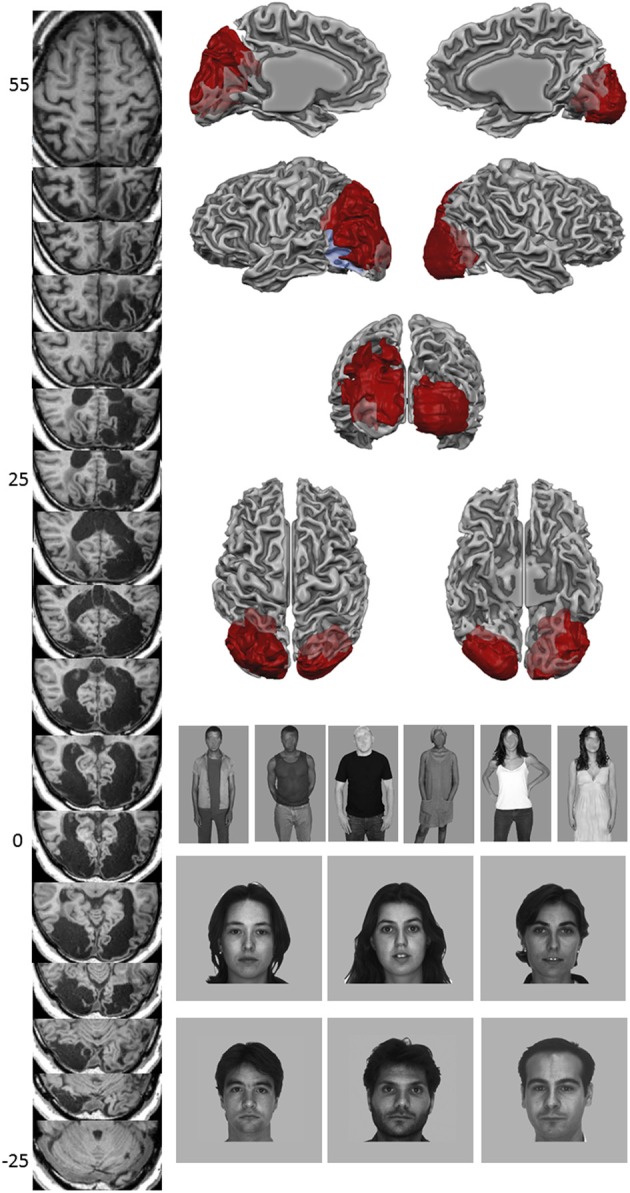
**Structural imaging results and stimulus examples**. The left column shows a regular series of axial T1 scan slices. Coordinates refer to Talairach space. The top row right shows medial views of a cortical reconstruction of TN's left and right hemisphere, with lateral views underneath. Below, a posterior view is shown, with underneath a dorsal (left) and ventral (right) view of TN's cortical reconstruction of both hemispheres. Gyri are shown in light gray, sulci in dark gray, lesion in red, and ventricles in light blue. The bottom right panel shows stimulus examples of body and face stimuli.

We recently reported above chance recognition of the human body shape in patient TN (Van den Stock et al., in press), the only available case with residual vision following bilateral destruction of the visual cortices (Figure 1) and clinical blindness over the whole visual field (Pegna et al., 2005; de Gelder et al., 2008). Here, we extend on those findings and provide additional evidence on perceptual sensitivity for body perception using signal detection analysis of behavioral responses. We also address new questions of high theoretical relevance concerning the neural bases of biological categories perception with new methods and contrasts of neuroimaging data not reported previously with TN. The exceptional subject and research question prompted us to conduct a study that was rather explorative in nature and in which the theoretical considerations outweighed the methodological nuances at the time of scanning.

Cortical blindness provides a unique opportunity to investigate fundamental questions about our conscious perception that immediately and effortlessly lets us experience objects rather than basic and disconnected visual properties such as luminance and contrast. Higher-level visual areas along the inferior temporal cortex composing the “ventral stream” primarily sustain normal object perception and categorical representation. These extrastriate areas show selective responses for specific object categories of high biological relevance such as body shapes or parts (Downing et al., 2001; Orlov et al., 2010), and faces (Kanwisher et al., 1997). The main input to these ventral extra-striate areas originates in the primary visual cortex (V1), but other V1-independent pathways in the mammalian brain can send visual information to areas in the ventral stream through direct connections with the lateral geniculate nucleus of the thalamus and the pulvinar that receive visual information directly from the retina (Harting et al., 1991; Lyon et al., 2010). In fact, notwithstanding that V1 lesions lead to clinical blindness in the corresponding part of the visual field, several basic visual properties such as shape and orientation can still be processed, a phenomenon known as “blindsight” (Humphrey and Weiskrantz, 1967; Weiskrantz et al., 1974).

Recent neuroimaging findings in patients with unilateral V1 destruction reported extensive activations of extra-striate visual areas in the intact hemisphere in response to stimuli presented in the blind field (Goebel et al., 2001; Tamietto and de Gelder, 2010; Van den Stock et al., 2011), as well as the formation of new fiber connections linking spared subcortical areas in the damaged hemisphere with extra-striate areas in the intact hemisphere (Bridge et al., 2008; Tamietto et al., 2012). Therefore, this leaves open the possibility that preserved object categorization for unseen stimuli is, at least partly, mediated by striate and extra-striate visual areas along the ventral stream of the intact hemisphere indicating an interhemispheric compensatory mechanism. The present study offers the unprecedented opportunity to rule out this possibility, as the participant had bilateral lesions destroying visual areas in both hemispheres.

## Methods

### Participant

TN is a man who became cortically blind at the age of 52 following two consecutive strokes with a 36-day interval that destroyed his left and right visual cortices. His first stroke occurred in the left parieto-temporo-occipital area, producing right hemiplegia and transcortical sensory aphasia, which receded rapidly, in addition to a dense and persistent right homonymous hemianopia. The second hemorrhage subsequently occurred in the right occipital lobe causing the loss of his left visual field with no other signs of neuropsychological deficit (and in particular no behavioral signs of spatial neglect). Clinically, TN behaves as a blind person using a stick to track obstacles and requiring guidance by another person when walking around. Extensive behavioral and neuro-imaging experiments could not provide any evidence of perceptual awareness or functioning of primary visual cortex (Pegna et al., 2005; de Gelder et al., 2008). The current testing took place 7½ years after his cerebro-vascular accidents.

#### Lesion

The lesion in the left hemisphere includes most of the occipital lobe, with minimal sparing of the medial ventral part of the inferior occipital gyrus and anterior part of the lingual gyrus, which are however atrophic. The lesion extends anteriorly to the middle part of the fusiform gyrus leaving the parahippocampal gyrus grossly intact. Laterally, it extends to the medial inferior temporal gyrus. Dorsally, the hemorrhage reached the superior parietal lobule and spared the ventral part of the precuneus.

The right hemisphere lesion is smaller and includes most of the occipital lobe, with limited sparing of the medial part of the posterior lingual gyrus and medial part of precuneus, but, again, these spared areas appear atrophic. The anterior border stretches to the middle part of the fusiform gyrus and included the posterior inferior temporal gyrus, but spared the parahippocampal gyrus. The gray matter around the lesions appears largely disconnected from the white matter, and atrophy can be observed. Both lateral ventricles expanded, especially in the posterior part. No anatomic input to striate areas could be detected in either left or right hemisphere. Moreover, previous DTI and EEG analyses revealed that TN does not have input to V1 or any inter-hemispheric cortical transfer in the occipital cortex via the corpus callosum (Pegna et al., 2005; de Gelder et al., 2008). Figure 1 shows axial slices of TN's occipital cortex and a reconstruction of TN's cortex and lesion.

#### Visual perimetry

In order to document any possible changes in TN's visual field due to functional cerebral reorganization, a high-resolution visual perimetry was administered the day before the present experiment. Stimuli consisting of small white circles (1°; stimulus luminance 95 cd/m^2^) were presented against a dark background (2 cd/m^2^) on a 17-inch computer monitor. The stimuli were presented one at a time for 300 ms at each of 64 different positions (16 stimuli for each visual quadrant) with onset and offset signaled by two different sounds. The inter-stimulus interval was 3 s. TN was required to report verbally when any stimulus change was consciously detected. Emphasis was placed on the requirement to report “normal” conscious perception of a visual stimulus, as opposed to a “feeling” that a stimulus occurred, but without any definable and conscious visual perception. This procedure enabled us to map TN's visual field within an ideal grid spanning 24° of horizontal and 20° of vertical eccentricity from the central point for each visual quadrant, with 6° increments along the horizontal meridian and 5° increments along the vertical meridian, with the innermost stimuli at 3° or eccentricity. A visual perimetry was also performed with flickering, instead of static, stimuli. The same procedure as before was used, with the only exception that white circles were presented for 300 ms with a flickering rate of 20 Hz. Consistent with the case history and with previous examinations that used this identical procedure (de Gelder et al., 2008), TN showed clinical blindness in all portions of the visual field tested, with either static or flickering stimuli, as he did not report the presence of any stimulus. In the past, a visual perimetry has been also performed with bigger flickering dots subtending 3°, in addition to dots of 1° as in the present case, while TN was asked to point to these dots with the index finger. Lastly, TN's conscious visual abilities have been tested also in a forced-choice task with big circular patches of different spatial frequencies subtending either 26° or 32° and presented centrally for 2 s. In all these tests TN showed complete clinical blindness over the whole visual field.

### Stimuli

Images of faces, whole bodies (with faces blurred), butterflies, cars and pixelwise scrambled images were matched for size and luminance. All stimuli were fitted in a background frame that sustained a visual angle of 8° × 10.5° from a viewing distance of 50 cm from the screen and had a mean luminance of 15 cd/m^2^. The bodies and faces were displayed in frontal view with a neutral expression. The faces showed direct gaze and the bodies were clothed, with the conventional form-fitting of the clothes to optimize ecological validity. Each of the five categories consisted of 24 different items for a total of 120 images. Half of the bodies and half of the faces were female. Half of the bodies in the fMRI-study were Caucasian and half were black African (as TN hails from sub-Saharan Africa).

### Procedure

#### Behavioral object categorization

The behavioral experiment was divided in 5 blocks, each targeting one of the 5 stimulus categories, and lasting approximately 5 min. In each block, all 120 stimuli (24 stimuli for each of the 5 categories) were presented singly at the center of the screen for 1500 ms, their onset, and offset announced by two different sounds. In each block TN was required to “guess” verbally on a trial-by-trial basis and with no time constraint whether the projected stimulus belonged to the target category or not, while the target/non-target ratio was unbeknownst to him. For instance, if the target category in a given block was face, TN was asked to respond “yes” or “no” to indicate whether or not the projected stimulus was a face, and so on for the other blocks and target categories. The scrambled category was defined as a meaningless rectangle patch of the same dimensions of the other stimuli. Each response was followed by a rating in which TN indicated his confidence in his response on a 4-point scale (1 = “least confident”; 4 = “most confident”). The order of the targets to be guessed in the five blocks was the following: (1) Faces; (2) Scrambles; (3) Bodies; (4) Animals; (5) Objects. The blocks were alternated with rests lasting about 5 min. On 25% of the trials, and after his response to the main categorization task, TN was also asked randomly to report, using a yes/no response, whether he had consciously “seen” or not the stimulus or some of its features. We paid attention to clarify the distinction between the primary categorization task, in which TN was required to “guess” the category of the stimuli, although unseen, from this secondary task in which he was instead asked to report conscious visual experiences and not to guess. This secondary task provided the opportunity to further assess TN's visual awareness during the categorization task and for the same stimuli.

#### Signal detection theory analysis

Signal detection theory (SDT) considers decisions as depending on subject's perceptual sensitivity to differences between stimuli (*d*′), in the present case between the 5 different categories, as well as on subject's response criterion (or bias), which is the tendency to favor a response independently of sensitivity (e.g., the putative tendency to report a specific category). This distinction cannot be posed by simply comparing accuracy in detecting a given stimulus category, as normally done with a traditional approach that analyzes percentage of correct recognition with a binomial test. In fact, this latter traditional approach does not consider false alarms and would reflect sensitivity reliably only assuming the absence of any response bias. According to SDT, the performance in yes/no tasks is fully described by four parameters: hits, misses, correct rejections and false alarms. Hits refer to correct “yes” responses on signal trials; that is, on trials where the target stimulus category is displayed; whereas misses refer to incorrect “no” responses on signal trials. Correct rejections refer to correct “no” responses on noise trials; that is, on trials where the non-target stimulus categories are displayed; whereas false alarms refer to incorrect “yes” responses on noise trials. Rating tasks, as used here, are like yes/no tasks insofar as they present only one stimulus type during each trial, with the only exception that rating tasks require an additional graded response following the first dichotomous response. They are typically used to measure sensitivity. Because each response (“yes” or “no”) had four ratings associated with it, there were eight possible responses for each trial that can be graded from the most confident “no” response to the most confident “yes” response (Azzopardi and Cowey, 1997; Stanislaw and Todorov, 1999). TN's ratings were thus used to determine points on the receiver operating characteristics (ROCs) curve, which plots the hit rate as a function of false alarm rate for all possible values of the criterion and for each stimulus category independently. A rating task with 8 possible responses determines 7 points on the ROC curve, each corresponding to a different criterion, so that one criterion distinguishes rating of “1” from rating of “2,” and so on. The area under the ROC curve is a measure of sensitivity unaffected by response bias and can be interpreted as the proportion of times subjects would correctly identify the target signal if signal and noise were presented simultaneously. The ROC area typically ranges from about 0.5, meaning that signal cannot be distinguished from noise, to 1, meaning perfect performance. The 7 ROC points for each curve of the five stimulus categories were then transformed in *z* scores for each pair of hit and false alarm-rates, and sensitivity was measured as *d*_*a*_, a variant of *d*′ that takes into account non-unit slopes of z-transformed ROCs and is appropriate in case of unequal variance between distributions. Finally, differences between pairs of *d*_*a*_ values for each of the five stimulus categories, expressed as normal deviates, *Z*_*da*_, were contrasted against each other by a series of two-tailed paired-sample *t*-tests to assess statistically significant differences in sensitivity. This same approach has been used in the past to investigate differences in perceptual sensitivity for consciously unseen stimuli in patients with unilateral cortical blindness (Azzopardi and Cowey, 1997) and with hemi-spatial neglect (Ricci and Chatterjee, 2004; Tamietto et al., 2007).

#### Functional magnetic resonance imaging (fMRI)

During scanning TN was instructed to keep his eyes open and look straight ahead. A passive exposure paradigm was chosen to ensure that category-specific neural responses were unaffected by top-down factors such as action execution or button-press.

The visual stimulation protocol consisted of alternating fixation (24,000 ms) and stimulation (20,000 ms) blocks. During stimulation blocks, 10 stimuli of the same category were presented one by one for 1500 ms with an interstimulus interval (ISI) of 500 ms. The run was pseudo-randomized with four consecutive series, each containing one block of every stimulus category. So there were four blocks of every condition. MRI data were collected on a Siemens 3T Trio MRI scanner (Siemens Medical Solutions, Erlangen, Germany). An anatomical scan was acquired using a 3-D MPRAGE T1-weighted sequence (TR/TE/TI = 2.5 s/2.9 ms/1.1 s, FOV = 230 mm, matrix 256×256, slice thickness = 0.9 mm). A T2^*^-weighted GRE EPI sequence was applied for whole brain BOLD sensitive MRI (TR/TE/Flip = 2 s/30 ms/85°, FOV 220 mm, matrix 86 × 86, in plane resolution 2.5 × 2.5 mm, 32 contiguous 3 mm axial slices with 0.45 mm gap). Preprocessing of the functional data included slice scan-time correction (cubic spline interpolation), 3D motion correction (trilinear/sinc interpolation) and temporal filtering (high pass GLM-Fourier of 2 sines/cosines). Functional data were transferred into Talairach space. The statistical analysis was based on the General Linear Model, with each condition defined as a predictor. The threshold was set at *p* < 0.00001 (uncorrected). All reported contrasts are t-contrasts.

## Results

### Behavioral results

TN continuously reported no conscious visual experience for any of the stimuli in which he was asked about his visual awareness, and he did not acknowledge any sensation that could possibly be helpful to consciously discriminate the stimulus category or shape. TN reported only the sensation of changes in screen luminance when the stimuli were displayed. We therefore report here his performance in non-conscious categorization.

**Figure 2:**
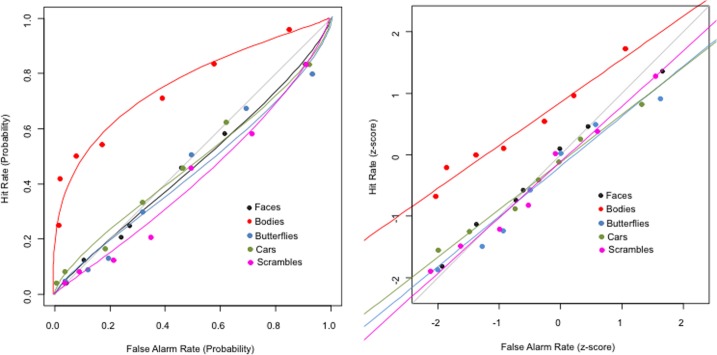
**Behavioral results**. On the left, 7-points ROC curves are shown fitted to TN's ratings as a function of the five stimulus categories reporting probability of hit rates vs. false alarm rates and on the right, Z-scores transformed ROC curves.

TN's responses to all stimulus categories as a function of the 8 possible ratings are reported in Table 1. Parameters of the 7-points ROC curves originating from TN's ratings for each of the five categories and the resultant graphs were computed with RscorePlus (Harvey, 2010) and are reported in Figure 2. ROCs were fitted to the data using a maximum-likelihood algorithm and the χ^2^ was used as goodness-of-fit measure. Results showed non-significant χ^2^ for all distributions, indicating a good fit between model and data (all *P*s ≥ 0.54). TN's perceptual sensitivity to human bodies was higher than to all other categories as shown by *d*_*a*_ values as well as by *A*_*z*_ values, the latter corresponding to the area under the ROC curve, which expresses sensitivity in terms of probability (*d*_*a*_ for bodies = 0.98, *d*_*a*_ for other categories ≤ −0.084; *A*_*z*_ for bodies = 0.76, *A*_*z*_ for other categories ≤ 0.48). This difference in the sensitivity for human bodies was significant compared to the sensitivities to the other four stimulus categories (all *P*s ≤ 0.004), which, in turn, did not differ from each other (all *P*s ≥ 0.54). The almost identical performance in all blocks that did not have bodies as targets indicate that the significantly better performance for bodies cannot be attributed to the order of presentation of the blocks or to fatigue effects in the other blocks^1^.

**Table 1:** **Responses to all stimulus categories and blocks as a function of the 8 possible ratings**.

**Block**	**Response**										
		**“No” non-target Confidence rating**					**“Yes” target Confidence rating**				
		**1**	**2**	**3**	**4**		**1**	**2**	**3**	**4**	
#1 Faces target	Stimulus type					Tot. misses					Tot. hits
	Target	5	3	6	4	18	1	2	2	1	6
						Tot. corr. reject.					Tot. false Al.
	Non-target	18	15	28	9	70	3	13	6	4	26
#2 Scrambles target	Stimulus type					Tot. misses					Tot. hits
	Target	6	3	6	4	19	2	1	1	1	5
						Tot. corr. reject.					Tot. false Al.
	Non-target	14	21	19	8	62	13	12	5	4	34
#3 Bodies target	Stimulus type					Tot. misses					Tot. hits
	Target	4	3	3	1	11	1	2	4	6	13
						Tot. corr. reject.					Tot. false Al.
	Non-target	21	18	26	14	79	9	5	1	2	17
#4 Animals target	Stimulus type					Tot. misses					Hits
	Target	5	4	3	5	17	4	1	1	1	7
						Tot. corr. reject.					Tot. false Al.
	Non-target	17	19	23	7	66	12	7	8	3	30
#5 Objects target	Stimulus type					Tot. misses					Tot. hits
	Target	3	4	5	4	16	4	2	1	1	8
						Tot. corr. reject.					Tot. false Al.
	Non-target	14	15	29	7	65	13	14	3	1	31

### fMRI results

First, we compared all objects vs. baseline to test whether there is any residual activity in early visual regions. The absence of any visual activity was reported in (de Gelder et al., 2008). This contrast was performed here at the threshold of *p* < 0.1 to assess possible trends indicating residual activity in striate or peri-striate visual areas indicative of functional reorganization occurring in the past 5 years. This threshold is in fact far more liberal than any reasonable statistical threshold and was applied here to test possible trends of activations that may go undetected with a conventional statistical approach. The results revealed no activation in early visual areas. Secondly, to test whether general object processing regions are present, we compared all objects vs. the scrambles, again at the threshold of *p* < 0.1. Again, this revealed no significant activations.

Considering TN's behavioral results of higher perceptual sensitivity and above chance discrimination of human bodies, and a previous report showing his residual visual capacities for processing facial expressions (Pegna et al., 2005), we performed two contrasts to assess neural structures involved in the non-conscious perception of biological categories such as human bodies and faces. We first compared bodies against butterflies, cars, and scrambles pooled together. Secondly, we compared faces against butterflies, cars, and scrambles pooled together.

**Figure 3:**
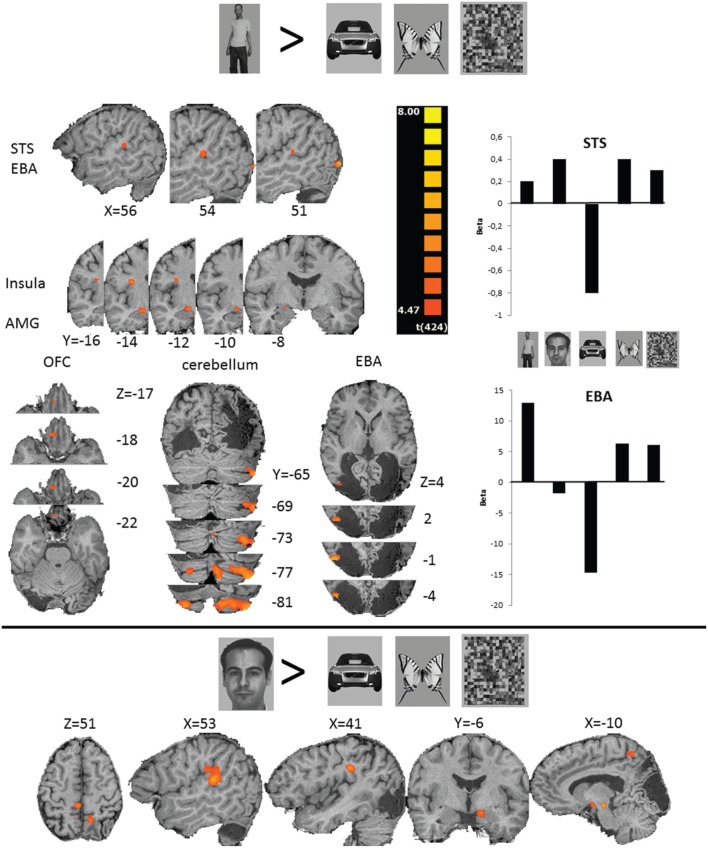
**fMRI results**. The top panel shows the activation clusters resulting from bodies vs. butterflies, cars, and scrambles (stimulus examples are shown on top). STS, superior temporal sulcus; EBA, extrastriate body area; AMG, amygdala; OFC, orbito-frontal cortex; superior temporal sulcus; EBA; insula and amygdala; cerebellum. Parameter estimates (Beta-values) for STS and EBA are shown on the right. The bottom panel shows the activation clusters resulting from faces vs. butterflies, cars, and scrambles (stimulus examples are shown on the left). From left to right: cingulate gyrus and superior parietal lobule; STS; supramarginal gyrus; amygdala; and periaqueductal gray. Coordinates refer to Talairach space.

Non-conscious perception of human bodies was associated with activity in a region of the ventral stream, anatomically corresponding in normal subjects to the right extrastriate body area (EBA), which is known to be selective for visual processing of the human body shape (Downing et al., 2001). While the extent of the lesions undoubtfully have an impact on the normalization procedure and hence any strict interpretation of coordinates should be done with caution, the activity we observe here falls largely within the normal range of EBA coordinates (Moro et al., 2008). In addition, bodies activated the right amygdala, orbitofrontal cortex (OFC), insula, superior temporal sulcus (STS) and bilateral cerebellum (Figure 3 and Table 2). The reverse contrast activated one large cluster containing most of the bilateral anterior temporal and orbitofrontal region. The areas activated when faces were presented include the right cingulate gyrus (CG), STS, supramarginal gyrus (SMG) and left superior parietal lobule (SPL), periaqueductal gray (PAG) and amygdala (AMG) (Figure 3). The reverse contrast activated primarily the bilateral orbitofrontal region and right lateral temporal cortex. Because activity in STS was reported for bodies as well as faces contrasts, we assessed the overlapping voxels of both clusters to further investigate the category specificity of the STS-activations. The results showed that 271 of the 338 voxels (80%) responding to bodies also responded to faces. We performed a *post-hoc* conjunction analysis to further argument the common activation of faces and bodies: bodies vs. (cars + butterflies + scrambles) ∩ faces vs. (cars + butterflies + scrambles). This again revealed activation in the right STS (see Table 3). To additionally substantiate the selectivity of EBA and STS responses for body perception, we used a split-half approach. We used the uneven blocks to localize EBA and STS and extracted the beta-values of the even blocks for all conditions. The results are displayed in Figure 3 reporting strong EBA selectivity for bodies, whereas STS appears less selective.

**Table 2:** **Activations for presentation of faces and bodies compared to butterflies, cars and scrambles**.

	**TAL**			**N(voxels)**	***t*-value**
	***X***	***Y***	***Z***		
**BODIES vs. (BUTTERFLIES, CARS, SCRAMBLES)**					
Ins	35	−14	24	142	4.932
STS	53	−29	10	149	4.778
EBA	46	−73	−1	609	5.573
AMG	22	−12	−8	222	4.975
OFC	7	32	−19	164	4.742
cerebellum	25	−81	−37	521	5.428
	−25	−77	−37	4098	5.235
**FACES vs. (BUTTERFLIES, CARS, SCRAMBLES)**					
SPL	−12	−53	53	345	4.853
CG	5	−36	49	419	4.903
SMG	42	−27	30	222	5.424
STS	52	−32	14	622	5.415
AMG	−11	−5	−11	307	4.827
PAG	−9	−20	−11	186	5.259

*X, Y, and Z refer to Talairach coordinates. Ins, Insula; STS, Superior Temporal Sulcus; EBA, Extrastriate Body Area; AMG, Amygdala; OFC, Orbitofrontal Cortex; SPL, Superior Parietal Lobule; CG, Cingulate Gyrus; SMG, Supramarginal Gyrus; PAG, Periaquaductal Gray*.

**Table 3:** ***Post-hoc* contrasts. X, Y and Z refer to Talairach coordinates**.

	**TAL**			**N(voxels)**	***t*-value**
	***X***	***Y***	***Z***		
**BODIES vs. SCRAMBLES**					
EBA	46	−73	0	463	5.100
**BODIES vs. (BUTTERFLIES, SCRAMBLES)**					
EBA	46	−73	0	622	5.462
**BODIES vs. (BUTTERFLIES, CARS, SCRAMBLES) ∩ FACES vs. (BUTTERFLIES, CARS, SCRAMBLES)**					
STS	53	−29	11	129	4.773
**BODIES vs. FACES**					
EBA	45	−71	−2	648	5.916
OFC	28	41	2	1225	5.633
LG	15	−61	1	355	5.658
ITG	60	−31	−19	365	5.566
**FACES vs. BODIES**					
TP	−24	−3	−23	16083	5.526
TP	34	7	−34	3895	5.635
AMG	13	0	−13	887	5.393
ITG	−56	−42	−13	866	5.374
preCun	6	−59	54	1532	5.199
SPL	−26	−48	58	793	5.410

Finally, we also directly compared bodies with faces. The regions responding more to bodies than to faces included the right EBA and orbitofrontal cortex, while the right amygdala, temporal pole, precuneus and a large cluster including the left temporal pole and amygdala responded more faces than to bodies.

## Discussion

The behavioral analysis support our previous report that the human visual system is able to process categorical properties of human body shapes despite total cortical blindness following bilateral destruction of V1 (Van den Stock et al., in press). The present results suggest that the body recognition effect cannot be attributed to a response bias, but reflects higher perceptual sensitivity to human bodies, as assessed by signal detection analysis. This indicates that categorical perception of human bodies does not rely, at least in the present case, on compensatory mechanisms from the contra-lesional hemisphere (Van den Stock et al., in press).

Neuro-anatomically, body stimuli activated the right EBA, AMG, STS, insula, OFC and bilateral cerebellum. The AMG has been primarily associated with processing of emotional faces (Breiter et al., 1996; Morris et al., 1996) and bodies (de Gelder et al., 2004) also in cortically blind patients (Morris et al., 2001), including TN (Pegna et al., 2005). There is however evidence that also neutral faces (Breiter et al., 1996) as well as neutral bodies (Van den Stock et al., 2014) trigger AMG activity. The present findings are compatible with these latter findings, as both neutral bodies and neutral faces activated the right and left AMG, respectively.

The EBA activation indicates that category-specific areas in the ventral stream can still receive visual input through a V1-independent pathway. Recent studies in humans and monkeys have consistently shown anatomical connections between LGN and a region of the lateral occipito-temporal cortex spatially overlapping with EBA, as well as between the same cortical area and the pulvinar (Sincich et al., 2004; Bridge et al., 2008; Schmid et al., 2010). This V1-independent visual pathway has proved critical for the non-conscious perception of unseen stimuli in cases of unilateral cortical blindness (Tamietto and de Gelder, 2010). The hypothesis that TN's relatively preserved ability to categorize non-consciously the human body shape relies at least partly on the EBA activation is in line with studies reporting impaired body shape processing when EBA is virtually lesioned (Urgesi et al., 2004, 2007; Moro et al., 2008; Pitcher et al., 2012; Van Koningsbruggen et al., 2013).

While TN was able to accurately categorize body stimuli, his performance for faces was at chance. This result is in line with clinical evidence from neuro-degenerative disorders showing face and body processing dissociation (Van den Stock et al., 2012a, 2013). The primary areas that are associated with processing the face-shape are the fusiform face area (FFA) and the occipital face area (OFA) (Haxby and Gobbini, 2011). TN's inability to categorize faces may be due to the fact that these areas are lesioned. On the other hand, AMG and STS were activated by faces in TN and these areas have been implicated in processing changeable and socially salient aspects of faces as well as facial expressions of emotions, rather than the face shape *per se* (Bruce and Young, 1986; Haxby and Gobbini, 2011). In fact, deficits in the perception of the structural configuration of faces, as manifest in patients with prosopagnosia, typically follow bilateral damage in face-specific areas in the ventral stream like FFA, but not STS or amygdala (Gainotti and Marra, 2011), while recognition of facial expressions, in particular fear, is impaired subsequent to amygdala damage. Consistently, a previous study reported that when TN was asked to guess the emotional expressions of faces, AMG was activated and responses were above chance (Pegna et al., 2005). Our current observation does not conflict with the original findings as facial expressions in the present study were always neutral. TN was asked to guess which of the different categories of stimuli were presented, thus implicating category-specific cerebral areas that were damaged insofar as faces were concerned. By contrast, the ability to guess the emotional expressions within the face category remains clearly possible in TN thanks to the preservation of the AMG.

It is noteworthy that, exposure to body images triggered activity in a number of areas other than those involved in category representation. In healthy subjects passive observation of human bodies activates a broad range of brain structures (de Gelder et al., 2004). This was also the case in patient TN, as passive exposure to unseen body shapes activated areas implicated in the representation of body schema, like the OFC (Van den Stock et al., 2014), and areas related to mapping somatic changes and to interoception, such as the insula (Damasio, 1999; De Gelder, 2006; Craig, 2009). However, the largest activation cluster in response to body shape was located in the bilateral hemispheres of the cerebellum. Although research on categorical perception has primarily focused on extrastriate visual cortex, there is evidence documenting the involvement of the cerebellum in categorical perception of faces (Van den Stock et al., 2012b) but also of bodies (Van den Stock et al., in press), as well as in motor resonance and action preparation consequent upon perceiving real or inferred body movements and shapes (Gallagher and Frith, 2004; Sokolov et al., 2010, 2012).

These additional activations in non-visual areas shed new light on the possible neural mechanisms sustaining body shape categorization in the absence of V1 and visual awareness. In fact, a common function of the OFC, insula, and cerebellum is to participate in the representation of bodily states. It is therefore possible that TN unwittingly “senses” the somatic changes elicited by the exposure to body shapes and uses it as a guide to guess which stimulus category has been displayed. This conjecture is consistent with previous reports showing that patients with visual agnosia, unable to discriminate consciously between different line orientations or shapes, may nevertheless use kinesthetic and proprioceptive information from bodily movements to compensate partly for their visuo-perceptual deficits (Murphy et al., 1996). Alternatively, there is indirect evidence from behavioral studies on tool processing that the dorsal stream also contributes to categorical perception (Almeida et al., 2008). It is unclear, however, whether the dorsal stream processes indeed semantic properties of objects categories or is rather sensitive to low-level visual properties, such as elongated shapes, which in turn may be used as a cue to guide categorical decisions (Sakuraba et al., 2012). It could be argued that our results reflect primarily the processing of the coarse shape and size of the body stimuli, i.e., primarily oriented along the vertical axis, rather the body shape *per se*. To test this alternative explanation, one would either need to compare body images with similar vertically elongated objects (e.g., trees) or scrambles, or include body images that differ in visual properties (e.g., body parts, or bodies sitting down). This issue clearly deserves further investigation.

The right STS is the only region that responded to both faces and bodies. The involvement of this area in normal subjects for perceiving socially and biologically relevant stimuli is well documented as evidenced in studies and models of face (Haxby and Gobbini, 2011) and body (Giese and Poggio, 2003) perception. Furthermore, it has recently been reported that the STS has a cardinal role in processing social stimuli (Lahnakoski et al., 2012). Our present results underscore the role of STS in social perception and suggest that it receives V1-independent input, presumably through subcortical connections (Sokolov et al., 2012).

While bodies and faces activated areas that have been associated with normal body and face perception, the other stimulus categories activated the OFC and temporal cortices. These areas have been previously associated with early top-down processes in visual object recognition, particularly modulated by low spatial frequencies (Bar et al., 2006). Although rather speculative, these activations in TN may reflect a neural correlate of perceiving and interpreting stimuli for which there is no significant residual vision.

Another issue relates to the ethnicity of the stimuli. While in the fMRI-experiment all faces were white Caucasian, half of the bodies were Caucasian and the other half were black African. Although we can't exclude the possibility that the face response may have been weaker due to the ethnicity, the theoretical possibility seems unlikely because (1) an extensive literature shows that presentation of faces belonging to an ethnicity different from that of the observer still activate the core face areas and that the differences observed across in-group/out-group ethnicities involve areas related to emotional or social value of the faces, the latter not being category-specific and (2) TN studied outside Africa throughout his early adulthood and later worked for many years in Europe and was thus equally experienced at Caucasian and non-Caucasian faces. Related to this, in the behavioral experiment only Caucasian bodies and faces were used. This shows that TN's sensitivity for body shapes was higher than for other categories even if the bodies displayed were from another ethnicity. On the other hand, our study only targets generic body shape recognition and it is not clear that this level of body processing is influenced by stimulus-ethnicity.

In conclusion, the current findings suggest that spared activity in category-specific visual areas may be a necessary albeit not a sufficient condition to categorize visual stimuli non-consciously. Possibly, other areas involved in mapping somatic and motor states may prove critical.

### Conflict of interest statement

The authors declare that the research was conducted in the absence of any commercial or financial relationships that could be construed as a potential conflict of interest.

## Footnotes

^1^The responses were scored as target and non-target without further keeping record of which of the 4 non-target stimulus category was presented as distractor. Unfortunately, this prevents analyzing whether bodily stimuli were more often correctly rejected compared to the other stimuli.
